# Supplementary figures and images for: Activation of autophagy is required for clearance of mitochondrial ROS in patients with asthenozoospermia
Source: PeerJ. 2025 Feb 13;13:e18827. doi: 10.7717/peerj.18827 (PMC11830362; doi:10.7717/peerj.18827)

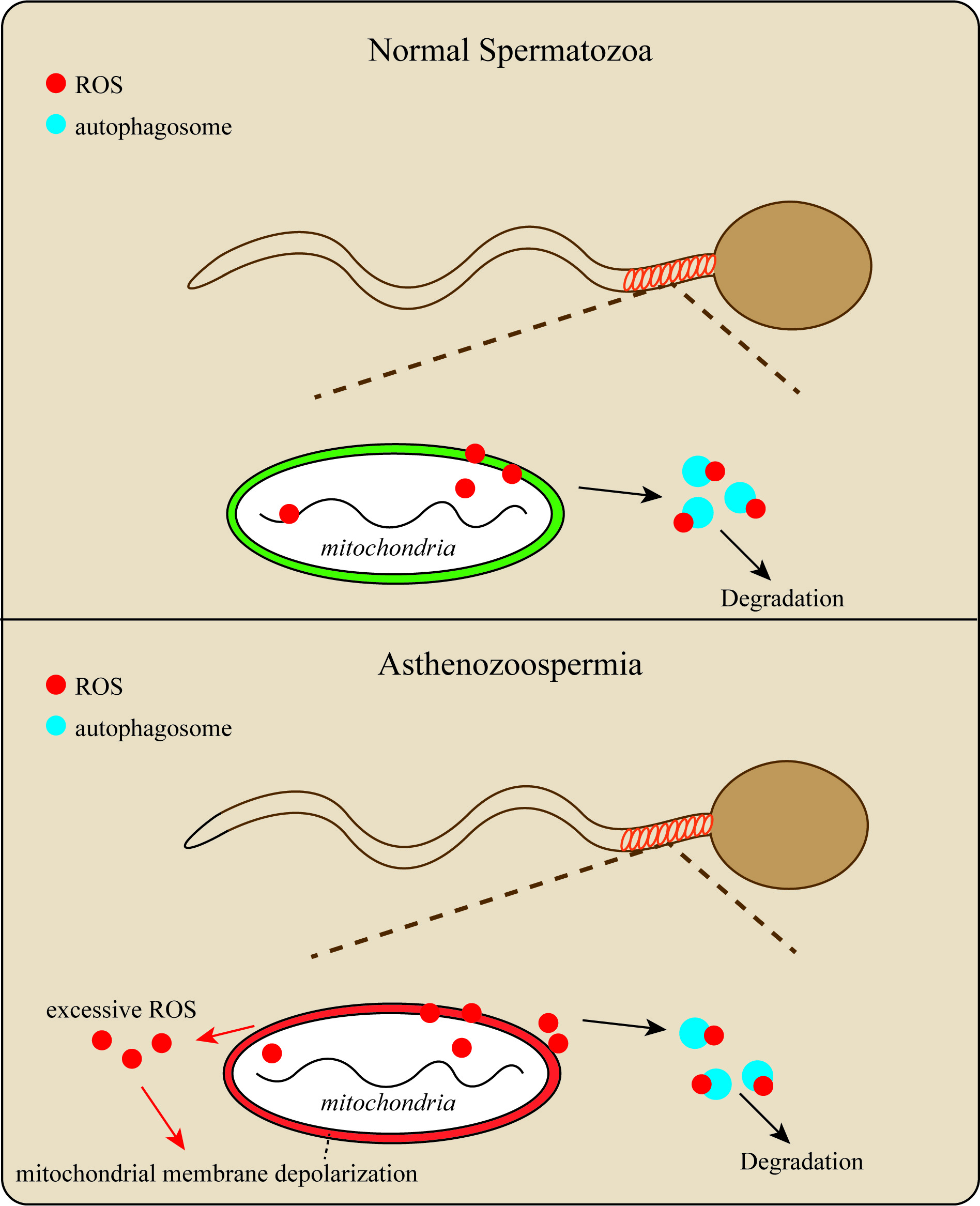

Supplement: Supplemental Information 1 — Upper Panel: In normal sperm, mitochondrial ROS can be cleared by autophagy, maintaining relative stability of the mitochondrial internal environment; Lower Panel: In asthenozoospermia patients, excessive ROS production by mitochondria exceeds the clearance capacity of autophagy, leading to mitochondrial membrane potential depolarization and mitochondrial dysfunction, ultimately resulting in sperm dysfunction [file peerj-13-18827-s001.jpg]

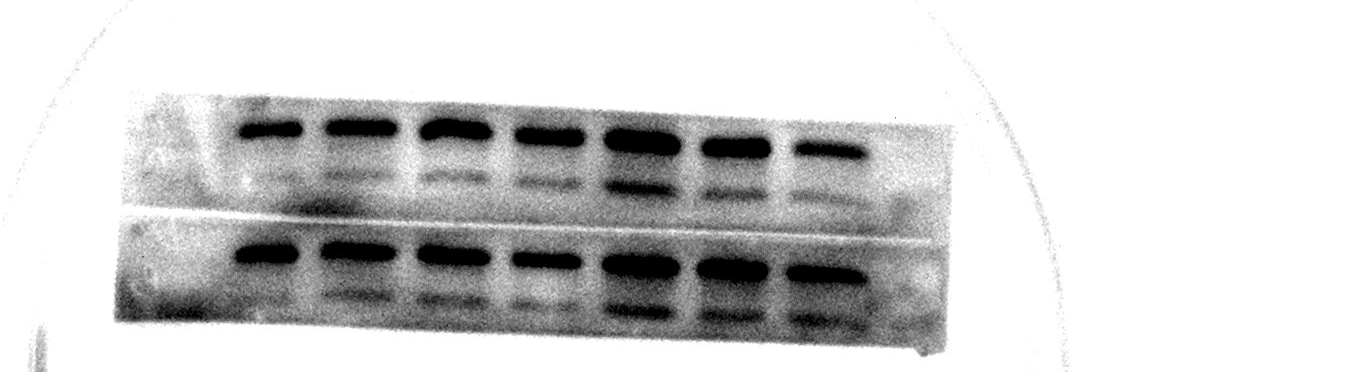

Supplement: Supplemental Information 4 [file peerj-13-18827-s004.zip › LC3-starve.jpg]

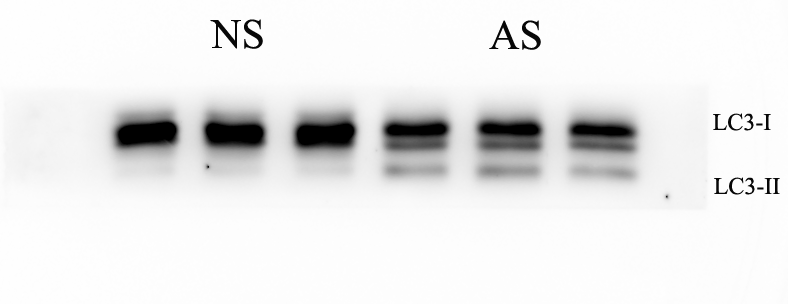

Supplement: Supplemental Information 4 [file peerj-13-18827-s004.zip › LC3.tif]

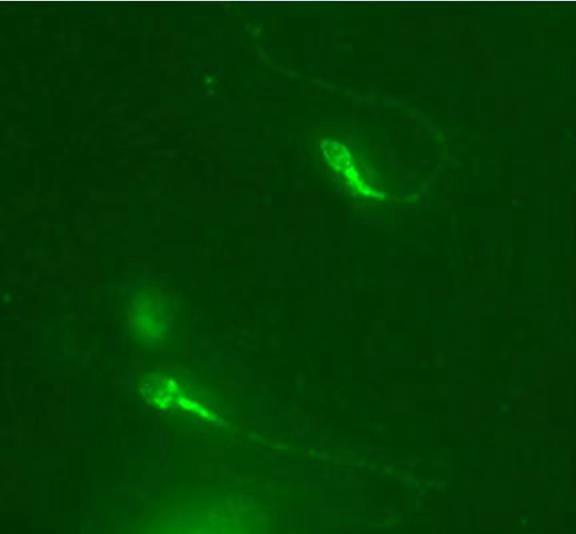

Supplement: Supplemental Information 4 [file peerj-13-18827-s004.zip › LC3-f.jpg]

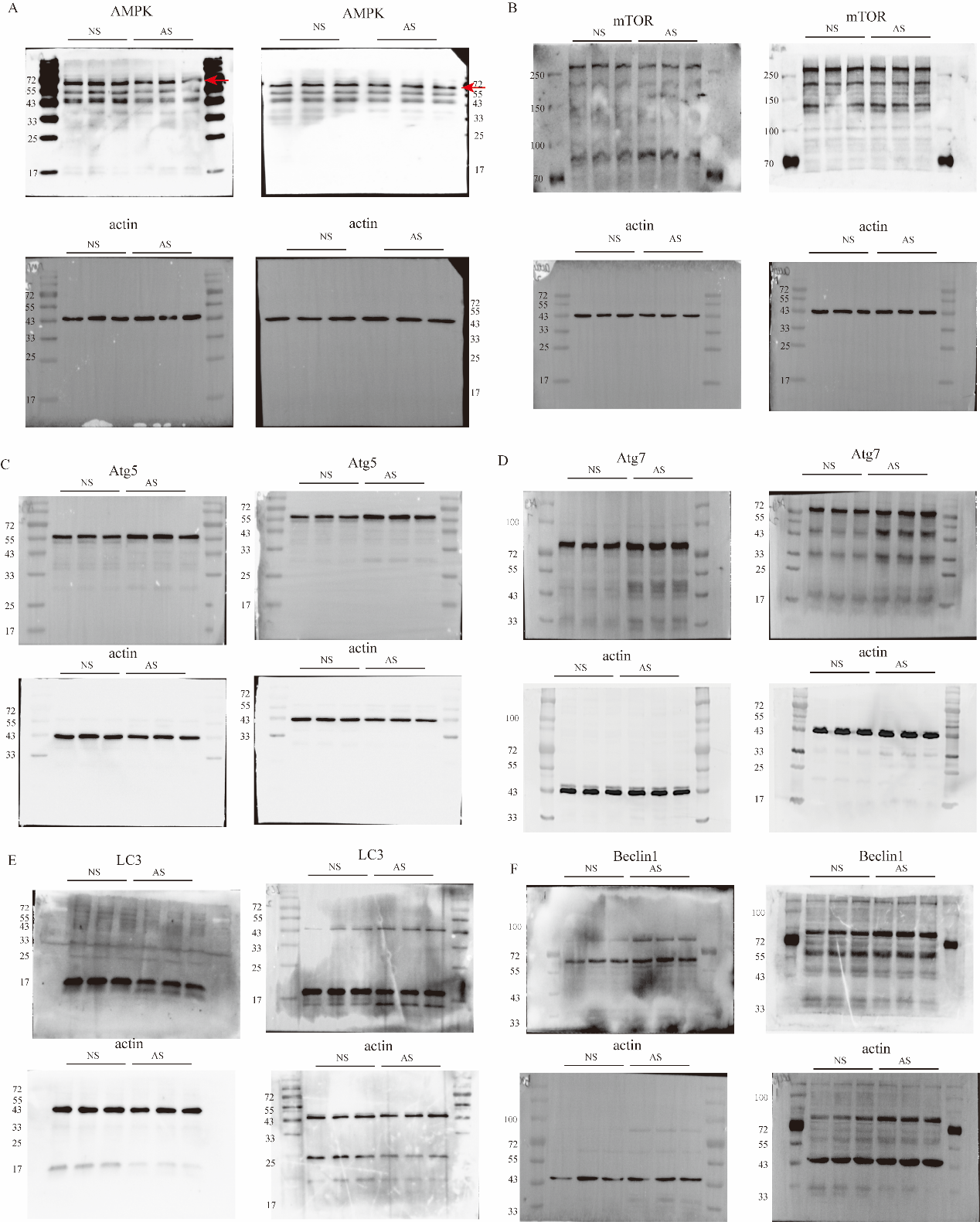

Supplement: Supplemental Information 6 [file peerj-13-18827-s006.docx]
